# The adaptation and validation of the satisfaction with stroke care questionnaire (Homesat) (SASC10-My™) for use in public primary healthcare facilities caring for long- term stroke survivors residing at home in the community

**DOI:** 10.1186/s12955-020-01450-9

**Published:** 2020-06-20

**Authors:** Aznida Firzah Abdul Aziz, Chai-Eng Tan, Mohd Fairuz Ali, Syed Mohamed Aljunid

**Affiliations:** 1 Department of Family Medicine, Faculty of Medicine, Universiti Kebangsaan Malaysia Medical Centre, Kuala Lumpur, Malaysia; 2 Community Health Department, Faculty of Medicine, Universiti Kebangsaan Malaysia Medical Centre, Kuala Lumpur, Malaysia; 3 Department of Health Policy and Management, Faculty of Public Health, Kuwait University, Kuwait City, Kuwait

**Keywords:** Patient satisfaction, Health care quality, Access, Evaluation, Stroke, Outpatient stroke facilities, Longer-term stroke

## Abstract

**Background:**

Satisfaction with post stroke services would assist stakeholders in addressing gaps in service delivery. Tools used to evaluate satisfaction with stroke care services need to be validated to match healthcare services provided in each country. Studies on satisfaction with post discharge stroke care delivery in low- and middle-income countries (LMIC) are scarce, despite knowledge that post stroke care delivery is fragmented and poorly coordinated. This study aims to modify and validate the HomeSat subscale of the Dutch Satisfaction with Stroke Care-19 (SASC-19) questionnaire for use in Malaysia and in countries with similar public healthcare services in the region.

**Methods:**

The HomeSat subscale of the Dutch SASC-19 questionnaire (11 items) underwent back-to-back translation to produce a Malay language version. Content validation was done by Family Medicine Specialists involved in community post-stroke care. Community social support services in the original questionnaire were substituted with equivalent local services to ensure contextual relevance. Internal consistency reliability was determined using Cronbach alpha. Exploratory factor analysis was done to validate the factor structure of the Malay version of the questionnaire (SASC10-My™). The SASC10-My™ was then tested on 175 post-stroke patients who were recruited at ten public primary care healthcentres across Peninsular Malaysia, in a trial-within a trial study.

**Results:**

One item from the original Dutch SASC19 (HomeSat) was dropped. Internal consistency for remaining 10 items was high (Cronbach alpha 0.830). Exploratory factor analysis showed the SASC10-My™ had 2 factors: discharge transition and social support services after discharge. The mean total score for SASC10-My™ was 10.74 (SD 7.33). Overall, only 18.2% were satisfied with outpatient stroke care services (SASC10-My™ score ≥ 20). Detailed analysis revealed only 10.9% of respondents were satisfied with discharge transition services, while only 40.9% were satisfied with support services after discharge.

**Conclusions:**

The SASC10-My™ questionnaire is a reliable and valid tool to measure caregiver or patient satisfaction with outpatient stroke care services in the Malaysian healthcare setting. Studies linking discharge protocol patterns and satisfaction with outpatient stroke care services should be conducted to improve care delivery and longer-term outcomes.

**Trial registration:**

No.: ACTRN12616001322426 (Registration Date: 21st September 2016.

## Background

Stroke care service delivery beyond the acute period is fragmented and challenging in most public healthcare systems worldwide [1, 2]. As far back as three decades, the lack of continuity of care between hospital and community was identified as a major problem which healthcare service providers should overcome to improve the outcome of stroke survivors in the community [3]. In order to enhance the patient centeredness of stroke care delivery, improvements will need to focus on the experiences of stroke survivors and their family caregivers as they move through diverse care environments [4].

Patients’ satisfaction and continuity of care have been recognized as important indicators of long-term care after stroke [5]. Hence, assessment of satisfaction with post-stroke care services beyond the acute phase is vital to ensure benefits of acute phase intervention are sustained to reduce stroke recurrence. In Malaysia, post- stroke care is delivered mainly at public primary care healthcentres led by Family Medicine Specialists (FMS), in outpatient primary care setting. However, this service is limited in terms of its outreach, catering only for patients who can access the healthcentres.

Data on satisfaction with post-stroke care services in this country is scarce. Tools to measure satisfaction with stroke care services have mostly been developed and validated in developed countries which have different public healthcare systems catering to the needs of stroke patients. One potential tool that could be relevant to measure satisfaction towards community post-stroke services is the Dutch Satisfaction with Stroke Care Questionnaire (SASC-19) HomeSat subscale [6]. The SASC has been validated for use in several countries, including the Netherlands [7], Germany [8], and Malawi [9] . Its strength lies in the fact that it measures satisfaction towards various healthcare-related services for post-stroke patients, in contrast with other tools which may measure other aspects such as life satisfaction or health-related quality of life. This study aimed to validate the English version of the Dutch Satisfaction with Stroke Care Questionnaire (SASC-19) outpatient subscale into Malay version, for use among Malaysian post stroke patients receiving longer-term stroke care at ten public primary healthcare facilities, in a trial- within-a- trial.

## Methods

### The questionnaire

Pound et al. first developed the Satisfaction with Stroke Care (SASC) questionnaire, a 13-item questionnaire to measure satisfaction with inpatient (Hospsat) and outpatient (Homesat) stroke care services in United Kingdom [6]. The questionnaire was then expanded to include a further 7 items to gather more details on satisfaction with stroke care after discharge [10]. This expanded version has been used in several studies and translated into Dutch as well (SASC19). The SASC was found to have good reliability for both scales, and demonstrated concurrent validity with the General Satisfaction Questionnaire, Barthel Index, Hospital Anxiety-Depression Scale and the SF-16 [11].

Patients were asked to indicate their agreement on each item using a 4-point Likert scale which ranges from 0 (strongly disagree) to 3 (strongly agree). The items can be analysed descriptively i.e. individually or summed up to produce a total score for all subscales. The higher the sum score indicated greater satisfaction [12, 13]. Based on the Dutch version, a patient who scores 22 or higher on the Homesat, indicates satisfaction with the care received after discharge from hospital [11].

As the focus of our study is on satisfaction with post-discharge care services, we aimed to produce a Malay language version of the SASC19 Homesat subscale, which measures patients’ satisfaction with outpatient stroke care services.

### Translation

Two FMS’ who are native speakers of Malay and bilingual professionally (i.e. Malay and English) were enlisted to translate the English version of the Homesat component of SASC19 into Malay language (forward translation). Differences in the terms were reconciled through discussions and final consensus. This harmonized version was subsequently sent to another two different FMS who translated it back into English. Both FMS’ involved were competent users of both Malay and English language, and were native Malay speakers.

All language versions were then compared by the researcher with the original version to ensure conceptual and semantic equivalence. Modifications were made to provide local examples of healthcare services available in the community. In item number 3 of SASC19– ‘meals-on-wheels’ was removed as this service is not available for patients residing at home in the community.

The harmonized version was then pretested on 5 post-stroke patients and 5 caregivers of stroke patients (ages 20–62 years old). This was done to determine its comprehensibility, clarity as well as determine ease of use among local respondents. No further modifications were required after pre-testing was complete. The final version was then used for the validation and to determine the satisfaction with the current outpatient services provided at ten public primary care healthcentres representing different zones in Peninsular Malaysia.

### Data collection

This study was part of a larger cross-sectional study to evaluate the profile and outcomes of longer-term stroke care provision for patients residing at home in the community and receiving longer term stroke care from public primary care healthcentres and the cost effectiveness of a pioneer integrated post discharge stroke care service based in primary care [14, 15]. Respondents were patients and/or caregivers of stroke patients attending public primary healthcare clinic between July 2012 till June 2013. Multistage sampling methods were used to recruit patients or caregivers of stroke from ten public primary care healthcentres across Peninsular Malaysia, for this phase of the study. The inclusion criteria were (1) patients aged 18 years and above, (2) clinically diagnosed with stroke due to any cause by the treating physician, with or without radiological confirmation. (3) Patients must have completed acute stroke treatment, discharged from hospital and referred or receiving treatment for long term stroke care at community healthcentres. Patients who were diagnosed with Transient Ischaemic Attack (TIA), or presented with isolated nerve palsy or those who were depressed were excluded from the study. The methodology is described in detail in another publication [14]. The validation of SASC19 was conducted during recruitment stage of the trial, and the respondents were asked to recall their satisfaction with public outpatient healthcare services received after discharge from a tertiary hospital i.e. mainly at the public primary care healthcentres. Patients or caregivers filled out the SASC19 questionnaires while waiting to see the Family Medicine Specialist. A trained research assistant was available to help answer any questions if required. The SASC19 questionnaire was distributed together with another self-administered questionnaire. For this validation study, an arbitrary estimate of 10 samples for each variable was estimated, requiring at least 110 samples for validation.

**Fig. 1:**
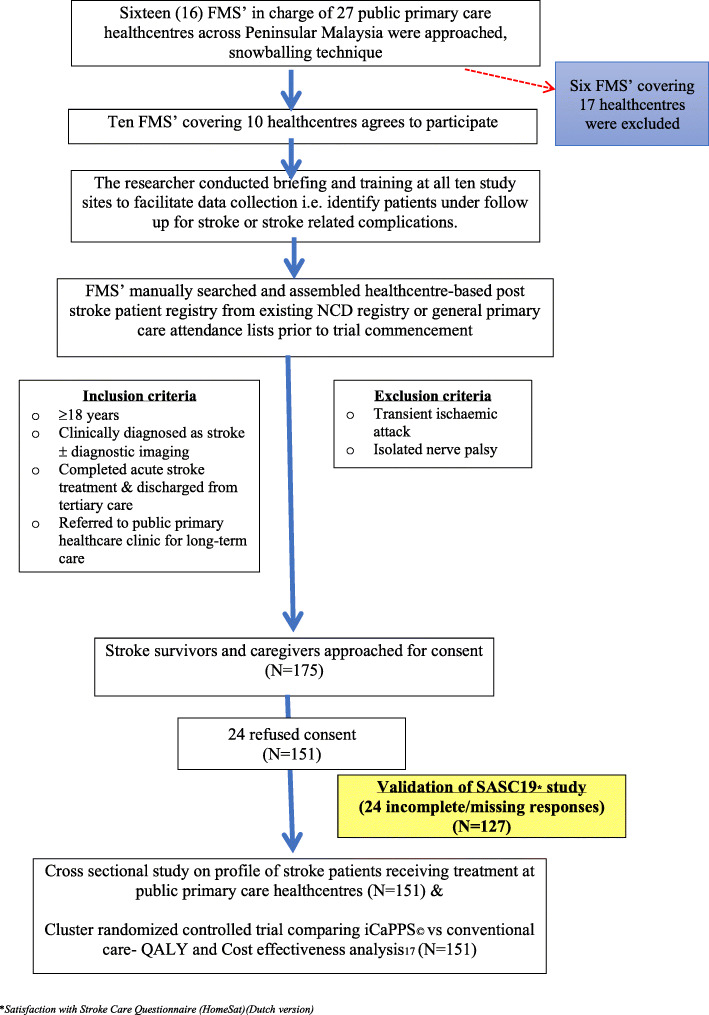
Study flow chart

Subsequently, the scores for satisfaction with outpatient stroke care services were then calculated using the finalized, validated version of the SASC10-My™ questionnaire. Refer Fig. 1.

### Data analysis

Descriptive statistics was used to analyse patients’ characteristics.

Samples with incomplete data were not included for analysis.

### Psychometric evaluation

The *feasibility* of the questionnaire was determined by the response rate, the time taken to fill the questionnaire and percentage of missing values per item.

*Internal consistency reliability* of the scale was determined using Cronbach alpha coefficient. An alpha coefficient value of 0.7–0.95 indicated good internal consistency reliability [16].

*Construct validity* was determined using the exploratory factor analysis. Suitability of the data for exploratory factor analysis was determined using the Kaiser-Meyer-Olkin coefficient and the Bartlett’s test of sphericity. The number of factors to extract was determined using both the Scree plot and Monte Carlo Parallel Analysis. We postulated that domains from satisfaction with post-stroke care services would be partly related and thus used Oblimin rotation in exploratory factor analysis. Items with factor loading above 0.40 would be retained. The internal consistency reliability for identified factors would be determined individually.

## Results

Altogether 175 participants were approached for this study. At recruitment, 24 patients/caregivers refused consent. The questionnaire was self-administered, filled by either the patient or the caregiver during the trial. After enrolment, another 24 respondents were excluded due to missing data, leaving only 127 (72.5%) respondents were included in the final analysis. The main reason for not being able to complete the questionnaires were: some questions were not applicable to them to respond to i.e. patients were never admitted for the acute stroke episode and hence could not relate to preparation of home settings after their discharge from hospital, nor did they receive any information about allowances or services before discharge from hospital, and could not relate to feeling neglected since discharge from hospital. The demographic profile of the study respondents is listed in Table 1.

**Table 1:** Baseline sociodemographic characteristics (*N* = 127)

Sociodemographic characteristics	Mean (sd)	n (%)
**Gender**		
Male		71 (55.9)
Female		56 (44.1)
**Type of stroke**		
Ischaemic		91 (71.7)
Haemorrhagic		10 (7.9)
Unspecified		18 (14.2)
Others		3 (2.4)
Not mentioned		5 (3.9)
Duration of stroke (years)	4.8 (5.4)	
Age at stroke diagnosis	55.4 (9.4)	

The average time taken by respondents to complete the questionnaire was ranged between 10 and 15 min.

The Kaiser-Meyer-Olkin coefficient for this dataset was 0.816 and Bartlett’s test of sphericity was significant (< 0.001). This shows that the data was suitable for exploratory factor analysis.

**Fig. 2:**
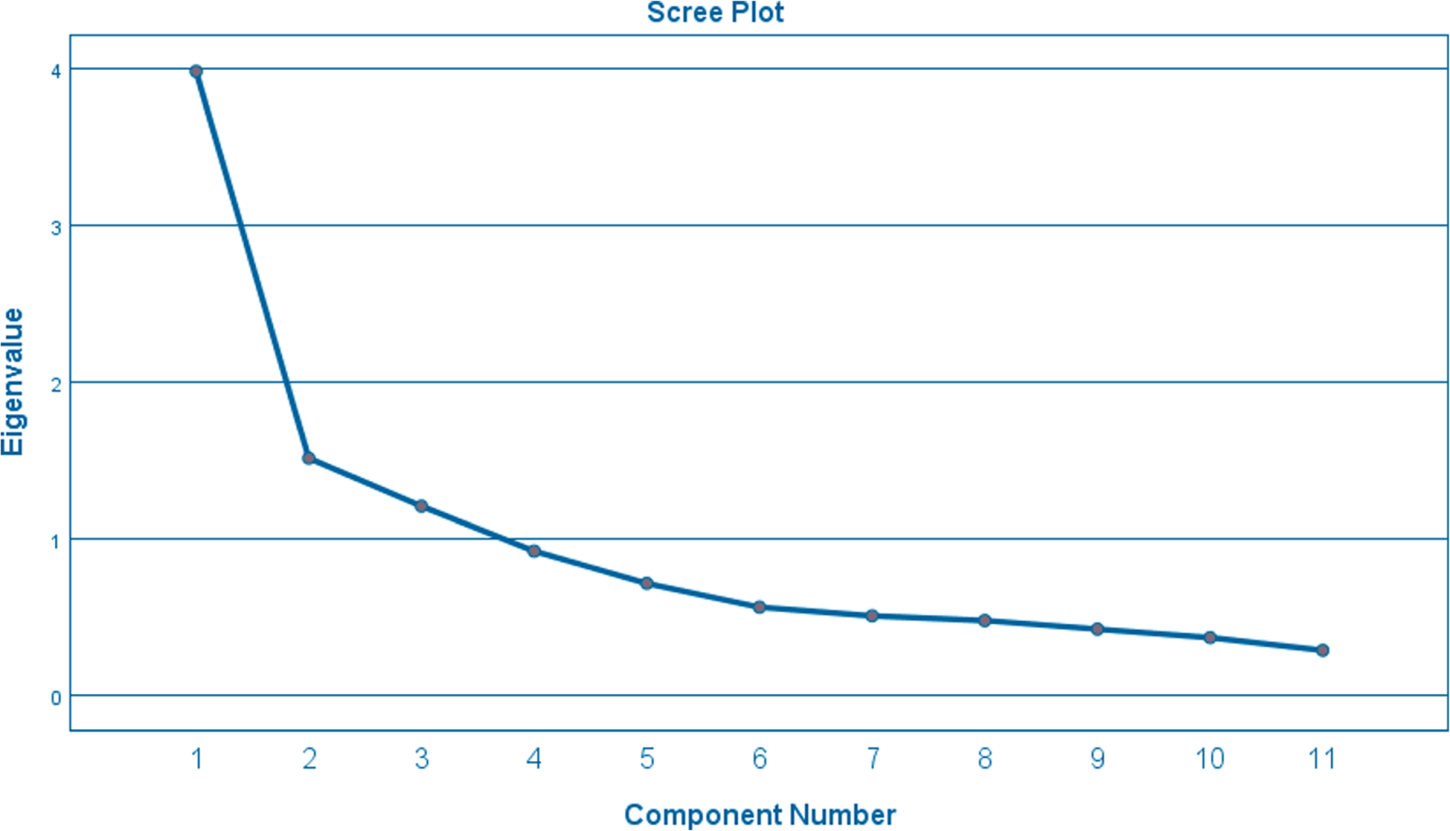
Scree plot

We used parallel analysis (refer Table 2) and Scree plot (refer Fig. 2) to determine the number of factors to retain for factor analysis. Although parallel analysis indicated that 3 factors should be retained, the elbow of the Scree plot indicated that only 2 factors should be retained. Taking into consideration the previous factor structure of the SASC19 HomeSat, only 2 factors were retained to ensure that the translated version would be comprehensible and interpretable compared to the original version [17]. Kaiser’s criterion is another factor retention method which states that factors with Eigenvalue of more than 1 is to be retained. However, Kaiser’s criterion has been found to over- or under-estimate the number of factors to be retained [17] and thus was not used here.

**Table 2:** Total variance explained and parallel analysis

Component	Initial Eigenvalues			Parallel analysis Random Eigenvalue	Comment
	Total	% of variance	Cumulative %		
1	3.987	36.248	36.248	1.5293	Retain
2	1.517	13.787	50.035	1.3656	Retain
3	1.210	11.001	61.036	1.2521	Retain
4	0.924	8.402	69.439	1.1516	Drop

Table 3 shows the factor structure following Oblimin rotation into 2 factors. One item (SASC8) had to be dropped as it failed to load into either factor. By removing SASC8, the Cronbach alpha value also improved to 0.830. This further supports that SASC8 should not be retained in the scale. When qualitatively examined, the item was about feeling neglected after leaving the hospital. This was something that was uncommonly expressed among locals, due to the collectivist culture of Malaysians in general [18]. Following discussion, the team agreed to drop item SASC8 from the overall questionnaire.

**Table 3:** Pattern matrix after Oblimin rotation

No	Items	Corrected item-total correlation	Alpha if item deleted	Component		Mean (sd)
				1	2	
**Satisfaction with support after discharge (Cronbach’s α = 0.820)**						
SASC9	I have had enough emotional support since I left hospital	0.580	0.794	**.819**	−.111	1.69 (0.68)
SASC7	Somebody has really listened and understood my needs and problems since I left hospital	0.595	0.791	**.815**	−.0.83	1.70 (0.64)
SASC5	I am satisfied with the practical help I have received since I left hospital	0.691	0.767	**.737**	.176	1.61 (0.83)
SASC6	I have received enough information about recovery and rehabilitation after stroke	0.684	0.768	**.664**	.294	1.57 (0.74)
SASC11	I know whom to contact if I have problems relating to my stroke	0.497	0.810	**.538**	.195	1.73 (0.82)
SASC4	I am satisfied with the outpatient services provided by the hospital (e.g. day hospital or appointment with doctors or therapists).	0.494	0.814	**.468**	.448	1.86 (0.78)
**Satisfaction with discharge transition (Cronbach’s α = 0.655)**						**Mean (sd)**
SASC1	I was given all the information I needed about allowances or services, I might need after leaving hospital (e. g. district nursing, medical social welfare officer, National Stroke Association or NASAM).	0.524	0.521	−.068	**.851**	1.14 (0.88)
SASC3	I get all the support I need from services such as district nursing etc.	0.536	0.536	−.044	**.819**	1.06 (0.85)
SASC2	Things were well prepared for my return home (i.e. aids such as a wheelchair or adaptations in my house, like grab handles in the lavatory or the shower had been organized.	0.366	0.633	−.040	**.524**	1.30 (0.93)
SASC10	I have received enough special equipment (e.g. wheelchair, commode, adaptations in my house like grab handles in the lavatory or the shower)	0.354	0.638	.183	**0.455**	1.11 (0.83)
**Dropped items**						
SASC8	I have felt neglected since I left hospital			−.363	0.101	1.16 (0.77)

The first factor was relevant to patient or caregiver satisfaction with support services after discharge from the hospital. It included items related to emotional and informational support, including having a reference contact in case of any new problems arising.

The second factor was regarding the patient or caregiver satisfaction with discharge transition services.

### Satisfaction with post stroke care services

The mean score for the 10-item SASCMy10TM was 15.74 (SD4.48). Overall, only 18.2% (22/121) were satisfied with outpatient post stroke care services. Table 3 shows the mean scores for each factor and the proportions of satisfaction towards each component. Refer Table 4 for details on patient satisfaction scoring.

**Table 4:** Patient and caregiver satisfaction towards post-stroke care services

Satisfaction level (***N*** = 127)	Mean score (SD)	Satisfied with care n(%)
Satisfaction with support services after discharge	11.0 (3.04)	52 (40.9)
Satisfaction with discharge transition services	4.79 (2.28)	14 (10.9)

## Discussion

### Summary of main findings

This study produced a reliable scale to measure Malaysian stroke patients’ or caregivers’ satisfaction towards post-stroke care services, which consisted of only 10 items compared to the original HomeSat component of the SASC19. The new scale, SASC10-My™ had two factors which measured satisfaction with support services after discharge and satisfaction with discharge transition services.

Item SASC8 (i.e. “I have felt neglected since I left hospital”) was dropped after considering its factor loading, internal consistency of the scale and its qualitative meaning. Feeling neglected could be unsuitable for use in the local sociocultural context. Malaysia has a collectivist culture where family support and cohesiveness during illness is something expected [18]. Among East Asian culture, the practice of filial respect and care to parents is seen as an obligation of adult children [19]. Respondents may have interpreted the feeling of being neglected as neglect from family members rather than from healthcare professionals. Neglect by family members could be interpreted as a social taboo as it is considered something shameful and could bring dishonour to the family [20], making patients or caregivers reluctant to disclose such feelings of neglect. Hence, their responses to this item would no longer be appropriate to represent their satisfaction towards stroke care services.

The two factors of the SASC10-My™ would be better for determining patient and caregiver satisfaction towards community-level stroke services as it has further defined satisfaction into the transition care services and post-discharge services. The overall Cronbach alpha coefficient was 0.830. Provision of transition care services and post-discharge services are not fully integrated in the local healthcare system. Transition care services depended a lot on hospital discharge practices, whereas post-discharge services were largely independent from the hospital inpatient services. Post-discharge services could include hospital-based outpatient services, health clinic services, private community-based services or non-governmental organisations. Hence it will be important to determine the level of patient or caregiver satisfaction towards these two very different levels of care.

**Fig. 3:**
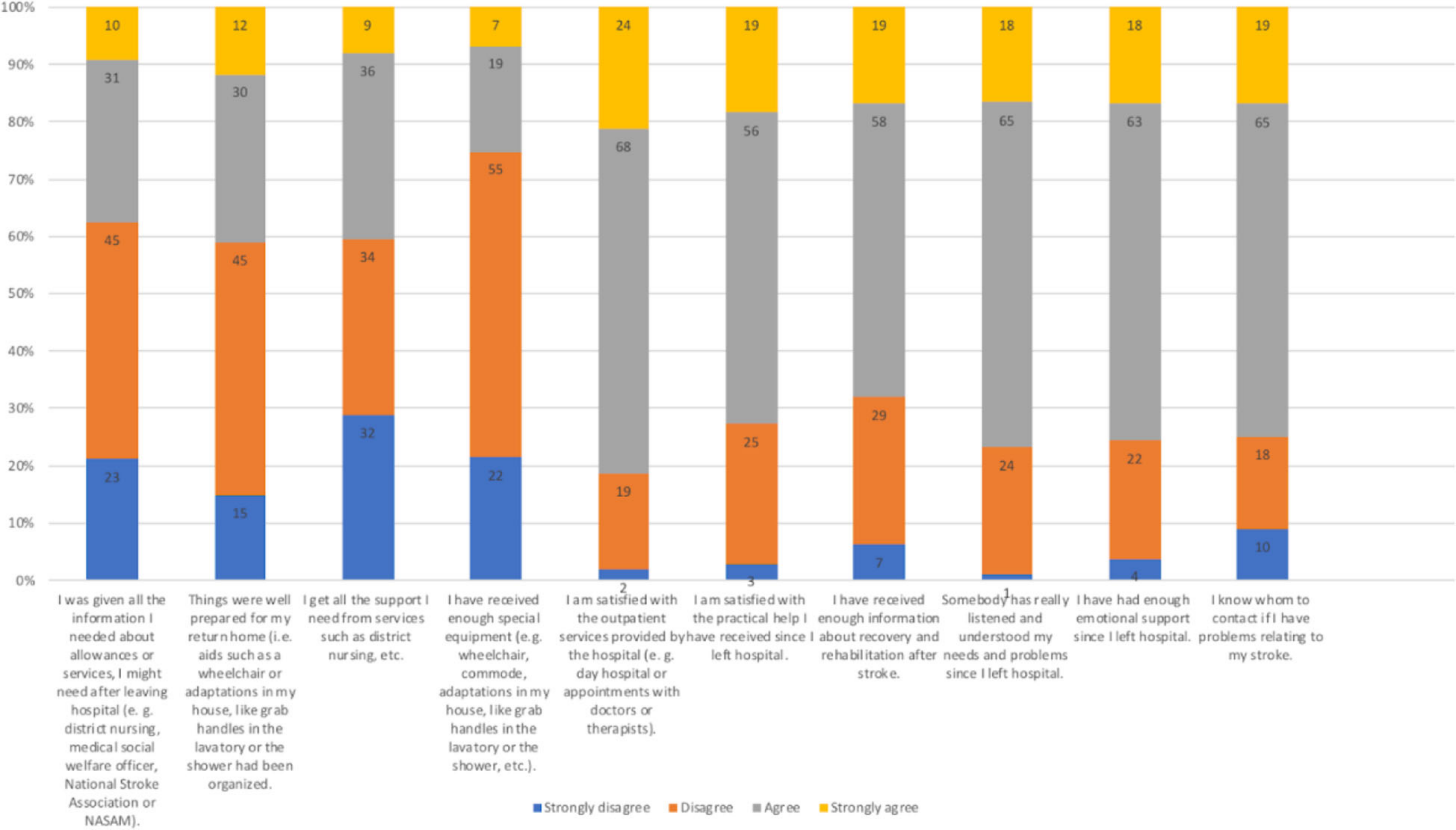
Components of satisfaction with 1 outpatient Stroke Care services at selected public primary care health centres in Peninsular Malaysia (*N* = 127)

Unfortunately, the overall response from post-stroke patients regarding the post-stroke care services was not favourable. Individual item responses showed that most respondents were not satisfied with provision of information regarding how to obtain rehabilitation equipment and supportive services upon discharge from hospital. This was also seen in their dissatisfaction regarding home preparations for discharge and support equipment (see Fig. 3). This reflected the overall process of discharge transition which deemed unsatisfactory. Gaps in discharge transition contributed to patients not receiving the support that they needed after discharge [14].

In contrast, satisfaction towards post-discharge services were better (40.9%). However, this still shows a great room for improvement in availability and accessibility to post-discharge services. In particular, respondents were not satisfied because they did not know who to contact in the event of new problems arising (see Fig. 2). Currently most hospitals do not provide contacts to a reference staff to patients in case of problems after discharge. Patients and caregivers are often told to seek help from the emergency department or nearest health clinic in case of problems. However, this was not practical in some instances when there are questions, which did not warrant a clinic or hospital visit. Examples of questions that may arise could include issues with medications or appointments. Ideally, an emergency contact person who could liaise with the primary team should be available to the patients. This could be a dedicated nurse or medical assistant who would know the patient’s background and provide guidance regarding what to do. Lack of an emergency contact could be due to insufficient resources. Providing the contact number of the ward or clinic could be helpful to provide support during office hours.

Provision of emotional support was satisfactory for most respondents. This could be because of the non-reliance of patients and caregivers on healthcare providers for emotional support. Again, a feature of collectivist culture, patients and caregivers tended to rely on their closer social support network of family members, neighbors and friends for emotional support. However, it might be good for healthcare providers to identify those with poor social support as these patients or caregivers may benefit from emotional support.

In studies conducted among patients who were discharged home to the community, patients and caregivers rated satisfaction with outpatient services ranged from 49 to 85% in European countries [4, 21, 22]. This highlights the fact that satisfaction with outpatient services is a challenge across most public healthcare services, regardless of either higher, low- or middle-income countries.

Post-stroke care delivery has been mostly haphazard [23] and suboptimal [24], particularly in countries which provide universal health coverage. As stroke is a continuing spectrum of complications resulting from multiple risk factors or chronic NCDs, the post discharge or longer-term care requires multiple interventions over long periods of time, delivered across different sectors. Hence, the need for subsequent management at community level to be coordinated and continued at appropriate care facilities once they are discharged.

### New and important aspects of this study

The post stroke or longer-term care has faced many challenges despite the advances in medical expertise and health technology. Ensuring optimal care to empower stroke survivors to cope with residual disabilities as well ensuring secondary prevention has been the focus of improving healthcare service delivery [3, 25, 26]. As such, the evaluation of satisfaction with healthcare services would provide vital information for all stakeholders on how best to reconfigure existing services to improve access as well as outcomes for stroke survivors and caregivers. To date, there has not been any studies in Malaysia which assessed satisfaction with post discharge stroke care services. Hence, the need to validate the questionnaire for use in the local healthcare system.

We believe our study is a first attempt to develop a tool to assess the Malaysian public outpatient stroke care services at community level in this country, using the SASC19 (Homesat) as reference. As the care for post stroke patients in Malaysia is largely fragmented and non-standardised for most parts of this country, it is necessary to have a tool which can be used as a benchmark with model stroke care services in developed countries for comparison.

The local scenario on post discharge stroke care services falls on the responsibility of the public primary healthcare services. The care delivery for post stroke patients provides an additional burden on the NCD work load at these healthcentres. Currently, post stroke care plans just focuses on provision of NCD care i.e. management of stroke risk factors, with minimal coordination of the rehabilitation aspects for a stroke survivor ([14, 27, 28]. Hence, the SASC10-My™ may be used by the primary healthcare team to identify areas for improvement in the current post stroke care service delivery at community level.

### Strengths and limitations of this study

#### Strengths

Patients enrolled for this trial were purposively recruited at baseline, from a healthcentre-based cluster randomised controlled trial involving ten public primary care healthcentres across Peninsular Malaysia. Respondents were at least 6 months after stroke, and resided at home.

The respondents for this study consist of post stroke patients and/or their caregivers who provided a wide range of perceptions on the services received.

#### Limitations

The recall bias in the responses provided by the patients or their caregivers are long term survivors in community dwelling. The location or institution whereby the respondents based their satisfaction with the outpatient treatment received after discharge from tertiary centre / acute treatment was not specified, i.e. public or private healthcare facility.

Missing data for some of the variables was unavoidable as community dwelling post discharge patients in Malaysia were inhomogeneous in terms of treatment received during and after stroke episode.

### Implications for future research

Confirmatory factor analysis can be done to determine whether the 2-factor construct is superior to the 3-factor construct.

Evaluation of post discharge stroke care service provision linked to the source of post discharge or longer-term stroke care accessed by the patients i.e. public or private healthcare facilities should also be undertaken.

## Conclusions

SASC10-My™ is a reliable and valid tool to measure caregiver or patient satisfaction with outpatient stroke care services in Malaysia. The management of post stroke services requires improvements to be made in areas of social support services after discharge and better discharge transition services or protocols.

## Data Availability

All data and materials are available on request from the author. Data and materials are stored at the Ethics and Research Secretariat of the Faculty of Medicine, National University of Malaysia (UKM), Kuala Lumpur, Malaysia.

## Abbreviations

SASC: Satisfaction with Stroke Care
LMIC: Low and Middle Income Countries
NCD: Non Communicable Disease
